# Nonlinear spin-wave excitations at low magnetic bias fields

**DOI:** 10.1038/ncomms9274

**Published:** 2015-09-16

**Authors:** Hans G. Bauer, Peter Majchrak, Torsten Kachel, Christian H. Back, Georg Woltersdorf

**Affiliations:** 1 Department of Physics, University of Regensburg, Universitätsstrasse 31, 93040 Regensburg, Germany; 2 Institut for Methods and Instrumentation in Synchrotron Radiation Research, Helmholtz-Center Berlin for Materials und Energy, Albert-Einstein-Str. 15, 12489 Berlin, Germany; 3 Institute of Physics, Martin Luther University Halle-Wittenberg, Von-Danckelmann-Platz 3, 06120 Halle, Germany

## Abstract

Nonlinear magnetization dynamics is essential for the operation of numerous spintronic devices ranging from magnetic memory to spin torque microwave generators. Examples are microwave-assisted switching of magnetic structures and the generation of spin currents at low bias fields by high-amplitude ferromagnetic resonance. Here we use X-ray magnetic circular dichroism to determine the number density of excited magnons in magnetically soft Ni_80_Fe_20_ thin films. Our data show that the common model of nonlinear ferromagnetic resonance is not adequate for the description of the nonlinear behaviour in the low magnetic field limit. Here we derive a model of parametric spin-wave excitation, which correctly predicts nonlinear threshold amplitudes and decay rates at high and at low magnetic bias fields. In fact, a series of critical spin-wave modes with fast oscillations of the amplitude and phase is found, generalizing the theory of parametric spin-wave excitation to large modulation amplitudes.

## Abstract (web summary)

Nonlinear magnetization dynamics underlie the operation of important spintronic devices. Here, the authors study NiFe thin films via X-ray magnetic circular dichroism, to develop a model for nonlinear spin-wave excitation by ferromagnetic resonance under small applied magnetic fields.

## Graphical abstract

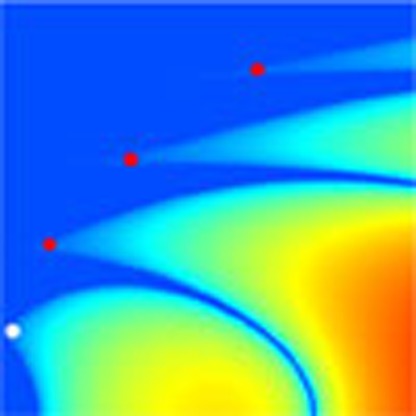

Nonlinear behaviour is observed in a vast range of physical systems. Although in some cases a transition from a well-behaved and predictable linear system to a nonlinear or even chaotic system is detrimental, nonlinear phenomena are of high interest, owing to their fundamental richness and complexity. In addition, a number of technologically useful processes rely on nonlinear phenomena. Examples are solitonic wave propagation^1^, high harmonic generation^2^, rectification and frequency mixing^3^. In many fields of physics, reaching from phonon dynamics to cosmology, anharmonic terms enrich the physical description but complicate the analysis. Often nonlinear effects can only be accounted for by performing cumbersome numerical three-dimensional lattice calculations or the physical description relies on dramatic simplifications. An analytic theory describing nonlinear phenomena would thus be highly desired.

The transition between harmonic and anharmonic behaviour usually occurs when an external driving force exceeds a well-defined threshold^4^. In the case of spin-wave excitations at ferromagnetic resonance (FMR) discussed in this study, the nonlinear spin-wave interaction depends on the amplitude of an external radio frequency (r.f.)-driving field and can thus be easily controlled. At large excitation amplitudes, one observes an instability of non-uniform spin-wave modes^5^. Moreover, large excitation amplitudes and thus nonlinear behaviour is also essential in the switching process of the magnetization vector (for example, in memory devices). In spintronics, the reversal of the magnetization in nanostructures is one of the key prerequisites for functional magnetic random access memory cells. Equally important is the understanding of spin transfer torque-driven nano-oscillators, which may function as radio frequency emitters or receivers. Both phenomena inherently involve large excitation amplitudes (and precession angles) of the magnetization vector deep in the nonlinear regime^6^,^7^,^8^,^9^,^10^,^11^,^12^.

In this study, we combine measurements of longitudinal^13^ and transverse^14^ components of the dynamic motion of the magnetization vector by X-ray magnetic circular dichroism (XMCD) as a function of r.f. power. At large driving amplitudes, our measurements clearly show that the low-field nonlinear resonance behaviour cannot be described adequately using existing models for nonlinear magnetic resonance^15^,^16^. To understand these data we develop a novel model that generalizes existing theories of spin-wave turbulence. We show that the basic assumption of a time-independent spin-wave amplitude parameter is not justified at low magnetic bias fields. In fact, pronounced fast oscillations of the amplitude and phase occur and dominate the nonlinear response.

## Results

### Experimental configuration

**Figure 1:**
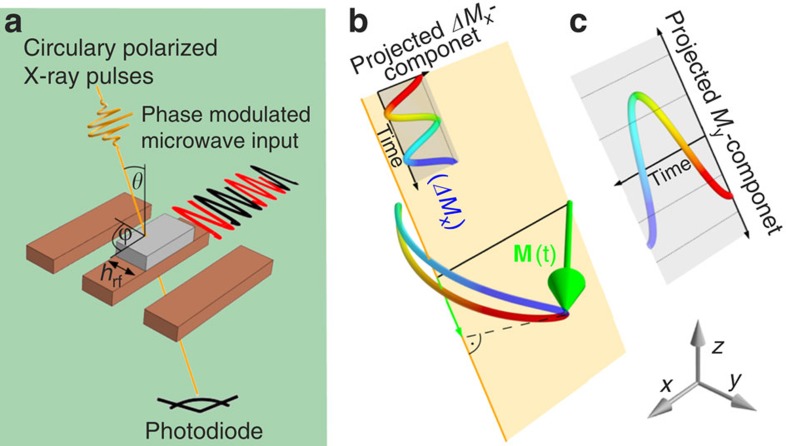
Experimental setup. (**a**) The X-ray intensity transmitted through the sample (grey) is modulated by the XMCD effect and detected by a photodiode. The XMCD signal is proportional to the magnetization component projected onto the X-ray beam direction. As indicated in **a**, the phase of the microwave signal supplied to the coplanar waveguide (CPW) is modulated by 180 degrees, and the polar as well as azimuthal angles of the sample (with respect to the X-ray beam direction) can be adjusted. The in-plane component of the dynamic magnetization (shown as green arrow in **b**) is measured in a time-resolved manner. The magnitude of the signal is given by the projection onto the X-ray beam direction and mostly proportional to *M*_*y*_(*t*), as shown in **c**. To determine the change in the longitudinal magnetization at FMR, the sample is tilted in the *x* direction with respect to the X-ray beam and the time-averaged change of the longitudinal magnetization component due to FMR 〈Δ*M*_*x*_〉 is measured, as illustrated in the upper inset of **b**. The colour scale for the time in **b** and **c** covers one precession period (*t*=0 is red and *t*=2*π* is blue).

**Figure 2:**
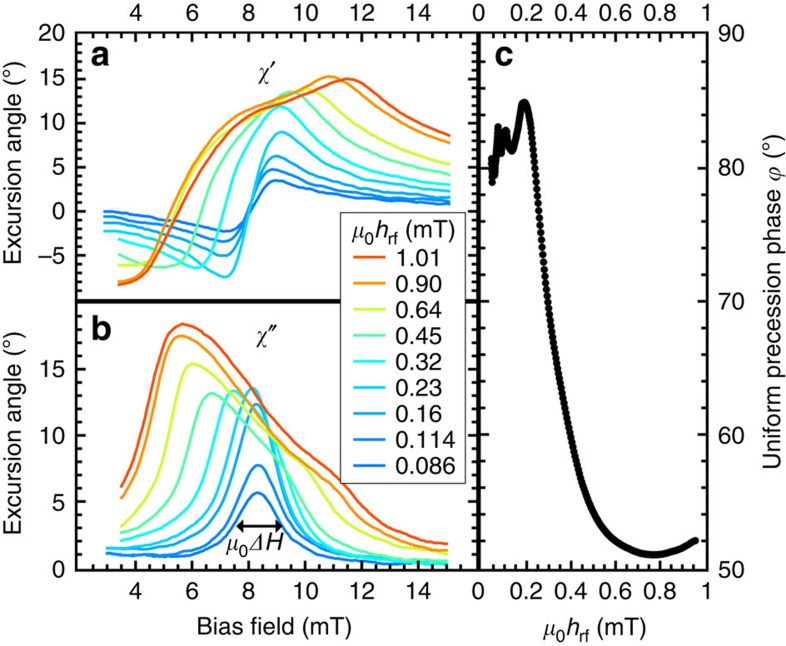
Time-resolved ferromagnetic resonance measurements at 2.5 GHz. (**a**) In-phase and (**b**) out-of-phase components of the normalized dynamic XMCD signal in units of degrees corresponding to the real and imaginary parts of the susceptibility. The maximum of the imaginary part of the susceptibility shifts to lower fields when a critical excitation level is reached^15^. Owing to the normalization procedure with static XMCD hysteresis loops, the absolute error for the excursion angles is below 5%. (**c**) From data as shown in **a** and **b**, the phase angle of magnetization with respect to the driving field is determined at the low amplitude FMR field, *H*_FMR_. The main mechanism limiting growth of the precession amplitude with an increasing driving field is a shift in the phase of the precessing magnetization. The instrumental limitations during these measurements lead to a phase error of up to 10°.

Our experiments are performed using Permalloy (Ni_80_Fe_20_) films deposited on top of the signal line of coplanar waveguide structures. In all measurements, a magnetic bias field **H**_B_ forces the static magnetization to be oriented in the *x* direction. A magnetic r.f. field oriented along the *y* direction leads to a forced precession of **M**, as illustrated in Fig. 1. The precession of the magnetization vector is strongly elliptical due to the demagnetizing field. As indicated in Fig. 1, the X-ray beam can be oriented at an angle *θ*=30° with respect to the film normal. In this geometry, the precession of the magnetization causes slight changes of the absorption of circularly polarized X-ray photons detected by a photo diode in transmission. In a first set of measurements, the X-ray beam is tilted in the *y* direction as illustrated in Fig. 1 (
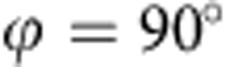
). A continuous wave r.f. excitation is synchronized to the X-ray flashes. Owing to the large ellipticity of the magnetization precession, the detected signal is mostly given by the in-plane magnetization *M*_*y*_ projected onto the X-ray beam direction. When the phase of the magnetic r.f. driving field is set to 90° or 0° with respect to the X-ray pulses, the measured signal represents either the real or the imaginary part (*χ*′ or *χ*′′) of the dynamic magnetic susceptibility 17 (cf. Fig. 2).

### Normalization of the XMCD signal

This measurement of the susceptibility is normalized to static hysteresis loops also measured by XMCD, as shown in Supplementary Figs 1 and 2 (setup and spectra, respectively). Thus, only non-thermal excitation of the magnetization is detected in units of *M*_s_. For the measurements shown in Fig. 2 the microwave phase and frequency (*ω*_p_=2*π*·2.5 GHz) are kept fixed, whereas for the resulting resonance curves the magnetic bias field **H**_B_ is swept for different amplitudes of the excitation field *h*_rf_. When the excitation field is increased above a critical amplitude of ∼0.2 mT, the main absorption shifts to lower fields. This effect is a consequence of the shift of the phase *φ* of the uniform mode above the threshold r.f. field. We find phase shifts of up to 35° at the small-angle resonance field *H*_FMR_ when the excitation amplitude is increased (Fig. 2c).

### Longitudinal component of the magnetization

Any magnetic excitation (coherent or incoherent) leads to a decrease of *M*_*x*_ of the order of *gμ*_B_, where *g* is the *g*-factor and *μ*_B_ the Bohr magneton. Therefore, to determine and separate coherent and incoherent components of the excitation we perform an additional measurement that is sensitive only to the longitudinal component of the magnetization vector *M*_*x*_. For this, the sample is tilted in the *x* direction (
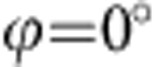
), the frequency of the r.f. signal is detuned by a few kHz from a multiple of the 500 MHz synchrotron repetition rate. In this way the phase information is averaged out and the experiment is only sensitive to the average longitudinal magnetization component. Lock-in detection in this case is achieved by amplitude modulation of the r.f. excitation. The corresponding signal is normalized again to static XMCD hysteresis loops.

On the one hand the measured decrease of the longitudinal magnetization component 〈Δ*M*_*x*_〉 is proportional to the density of non-thermal magnons *n*_*k*_ excited in the sample^18^. On the other hand, the population of the uniform mode *n*_0_=*n*_*k*=0_ can also be calculated from the time-resolved (coherent) measurement of *M*_*y*_ in the linear excitation regime. For this, the energy stored in the coherent magnon excitation Δ*E* can be written as:


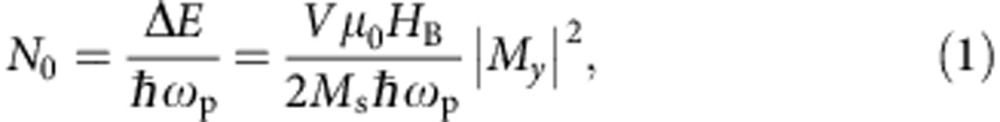


where *V* is the volume of the sample. The reduction of the longitudinal magnetization per magnon is found to be ∼5.5 *gμ*_B_. This large value is due to the highly elliptical precession 
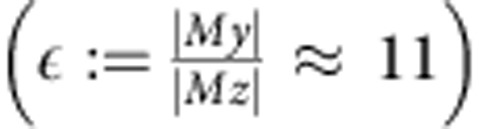
 and in agreement with 
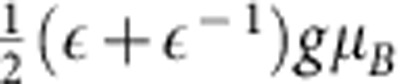
 expected from linear spin-wave theory.

**Figure 3:**
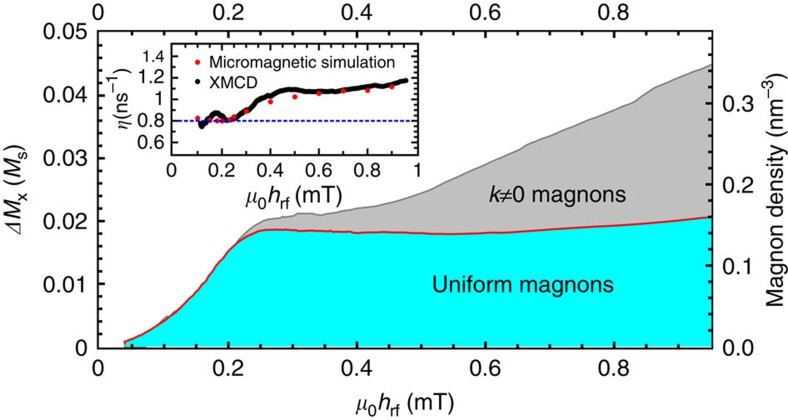
Number of excited magnons and magnon relaxation rate. The decrease of the longitudinal magnetization (Δ*M*_*x*_=*M*_s_−*M*_*x*_) with growing excitation amplitude measured for *H*_bias_≈*H*_FMR_. The blue line shows the measured Δ*M*_*x*_, which is proportional to the total number of magnons excited. Δ*M*_*x*_ corresponding to uniform precession (red line) is calculated from the coherent *M*_*y*_ components. Above the threshold, the total number of uniform magnons locks close to its threshold value (blue area), whereas the number of non-uniform magnons *n*_*k*≠0_ increases with *h*_rf_ (grey area). The inset shows the magnon relaxation rates that can be computed from these data. When a second-order Suhl instability process is assumed (*ω*_*k*_=*ω*_0_), the average relaxation rate *η* increases by ∼50%. Red points are obtained from corresponding micromagnetic simulations.

In Fig. 3, the driving field dependence of 〈Δ*M*_*x*_〉 from the time-averaged longitudinal XMCD–FMR experiment is shown and compared with the calculated 〈Δ*M*_*x*_〉 values obtained from the time-resolved transverse measurement of *M*_*y*_(t). It is noteworthy that the transverse component is only sensitive to *n*_0_ magnons. In the linear regime, both curves coincide and the excited magnon population only contains uniform *k*=0 magnons. Above a critical r.f. field of ∼0.2 mT, the two curves separate, owing to saturation of the uniform magnon occupation density *n*_0_ and the parametric excitation of higher *k* magnons in the nonlinear regime^5^,^19^,^20^, that is, the difference between the curves shown in Fig. 3 corresponds to the parametric excitation of additional *k*≠0 spin waves.

## Discussion

The saturation of the homogeneous mode population as a function of r.f. field amplitude (Fig. 3) is the consequence of an increased relaxation rate for this mode: the nonlinear coupling of the uniform mode to non-uniform spin waves opens additional relaxation channels. In this regime, the energy pumped into the homogeneous mode by the r.f. excitation is only partly relaxed by intrinsic uniform mode damping. In fact, a significant portion of the energy is distributed to non-uniform modes by additional magnon–magnon scattering processes and subsequently relaxed by intrinsic damping as illustrated in Supplementary Fig. 3. Conservation of energy requires that the energy pumped into the magnetic system is equal to the energy relaxed to the lattice by intrinsic Gilbert damping of the dynamic modes^21^:


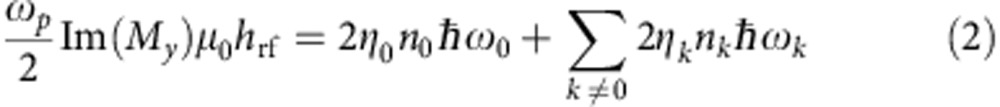


where *n*_0,*k*_ are the magnon densities, and *ħω*_0,*k*_ and *η*_0,*k*_ are the magnon energies and relaxation rates, respectively. At the FMR condition (*ω*_p_=*ω*_0_), only uniform magnons are directly pumped and all other magnons (with density *n*_*k*≠0_) are indirectly excited via nonlinear magnon–magnon processes. When we assume the latter to be second-order Suhl instability processes (*ω*_*k*_=*ω*_0_), we can calculate the expected magnon relaxation rate. The result of this is shown in the inset of Fig. 3 (dashed line). In the experiment, however, we find an increase of ∼50% for the average relaxation rate *η* compared with the relaxation rate of the uniform mode *η*_0_≈0.8 ns^−1^ at *ω*_p_=2*π*·2.5 GHz. This increased relaxation rate is unexpected, as microscopic theory of magnetic damping^22^ does not predict a wave vector dependence of the Gilbert damping constant *α* for the relatively small wave vectors that are relevant.

**Figure 4:**
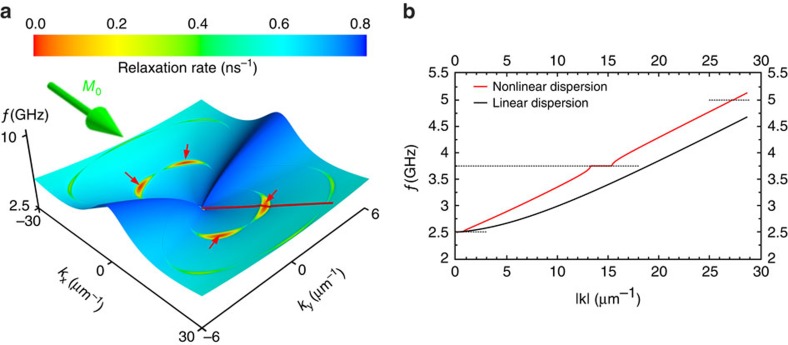
Spin-wave dispersion. (**a**) Precession frequency (*f*-axis) and relaxation rate (inverse lifetime of the spin waves shown as colour scale) as a function of the in-plane wave vector. The calculation is performed at an r.f. excitation frequency of 2.5 GHz with an amplitude slightly above the threshold field (*μ*_0_*h*_rf_=0.21 mT) at which the first pair of spin waves (**k**_crit._ ≈(±14.4,±1.7) μm^−1^) becomes unstable, as highlighted by four red arrows. The frequency of these spin waves has the largest spectral weight at 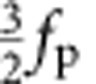. (**b**) Cross-sectional view of the spin-wave dispersion. As indicated by the red line in **a**, the cross-section is chosen in the direction of the critical spin waves. The nonlinearity causes frequency locking in the vicinity of the critical spin waves highlighted by the dashed lines and most clearly observed at 3.75 GHz. In addition, the spin-wave frequencies are shifted upwards by about 500 MHz relative to the dispersion in the linear regime.

**Figure 5:**
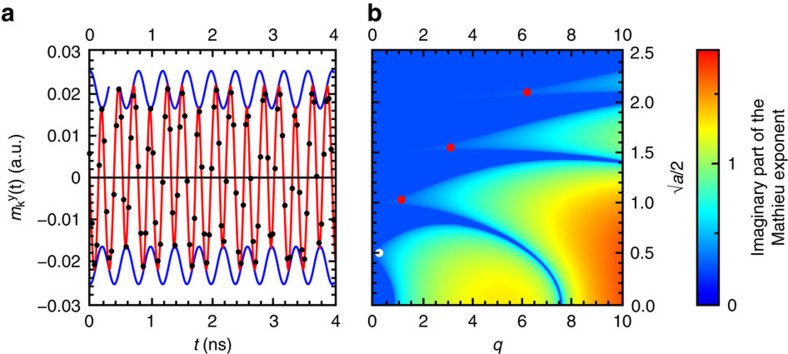
Time dependence of the critical spin-wave modes and instability diagram of the Mathieu equation. (**a**) Time dependence of the critical spin-wave modes from the Mathieu equation (red line). In agreement with micromagnetic simulations (points) we find that these modes are characterized by a significant modulation of the instantaneous resonance frequency and phase, as can be seen in the variations of the zero-crossing periods. The blue lines show the envelope of the amplitude oscillations. (**b**) The instability diagram of the Mathieu equation shows the relation between Suhl instability processes and the instability processes described in this study. The first- and second-order Suhl instability processes only represent the special case of vanishing modulation amplitude, which occurs at large bias fields. These processes are highlighted by the white point close to *q* ≈ 0. The new instability processes relevant at low bias fields are indicated by the three red points.

From extensive micromagnetic simulations, we found that the experimental data can in fact be reproduced quite well using a wave vector-independent damping parameter (red points in the inset of Fig. 3). This in turn implies that the physical explanation for the deviation from the Suhl model is to be found within the framework of the Landau–Lifshitz–Gilbert (LLG) equation. To unravel the physical origin for this behaviour, we develop a model based on the LLG equation that allows computing the properties of the critical spin waves in *k*-space in an efficient manner. This partially analytic approach can provide insight into the physics and reduces the computational effort drastically. We start from the LLG equation. Below the threshold excitation amplitude, only the uniform mode has a considerable amplitude. Therefore, we can restrict our considerations to linear terms in the *k*≠0 spin-wave amplitude *m*^*k*^. By algebraic transformations, one can show that the time evolution of *m*^*k*^ is then governed by a harmonic oscillator equation, which is parametrically driven by the uniform mode. In our case, this driving mostly occurs due to the dipolar fields *h*^*k*^, which strongly depend on the angle between the time-dependent uniform magnetization and the *k*-vector. A numerical time integration of the differential equation as a function of *k*-vector easily allows extracting the nonlinear dispersion and the decay rates for the spin waves in *k*-space. An example of this is shown in Fig. 4a. The calculation is performed for the parameters that correspond to the XMCD-FMR experiments and the excitation amplitude was chosen close to the instability threshold. The fundamentally new finding from our model is that the critical spin waves (inverse life times approaching zero) do not precess at the driving frequency as expected for the four-magnon scattering processes that usually lead to the second-order Suhl instability^23^,^24^,^25^. Instead, we find that the spin waves precess non-monochromatically at frequencies that are half-integer multiples of the driving frequency with additional oscillations of their amplitude and phase at the driving frequency (see Fig. 5a). In addition to the nonlinear shift of the spin-wave dispersion, we also observe a pronounced frequency locking effect to half-integer multiples of the driving frequency in the vicinity of the wave vectors for the critical spin waves (Fig. 4b).

To demonstrate more clearly that this nonlinear behaviour is fundamentally different from Suhl instabilities, we further simplify the numerical model. We assume that the time dependence of the potential term in the parametric oscillator equation 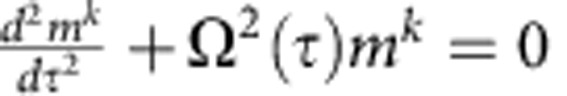 can be written as Ω^2^(*τ*)=*a*−2*q* cos(2*τ*), where the transformation of the time *t*→*τ* must be chosen to represent the instability process of interest (see further details in the Methods section). Although this assumption cuts off higher-frequency components, the simplified model is still able to reproduce our instability process as well as Suhl instability processes with the use of only two parameters (*a* and *q*). With these simplifications, the parametric oscillator equation assumes the form of the Mathieu equation (see Methods). Here 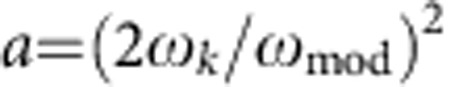 with *ω*_*k*_ equal to the mean frequency of the spin wave, *ω*_mod_ is the frequency of the modulation and the parameter *q* is the modulation strength. The instability diagram for this two-parameter equation is well known, owing to its significance for fundamental quantum mechanical problems^26^ and shown in Fig. 5b. By mapping the spin-wave instability processes onto this diagram, we find that although Suhl instabilities belong to the first instability region, the instabilities that we observe at low bias fields belong to the third instability region.

What distinguishes the first instability region from the others is that the modulation parameter is small (*q*/*a*<<1) and a perturbative approach can be used. In this case, the unperturbed states are harmonic oscillations with an amplitude that may only vary slowly in time. It is this assumption of a slowly varying envelope that breaks down for the higher instability regions and prevents the description of the instability processes that we observe by standard spin-wave instability theory. When the modulation amplitude (*q*/*a*) can no longer be considered small, the spin-wave precession also becomes considerably anharmonic, that is, higher Fourier components, separated by the frequency of the modulation, become important. This situation is mathematically identical to the motion of a quantum mechanical particle in a one-dimensional periodic potential when the kinetic energy becomes comparable to the potential height. Although for a quantum particle the amplitude and phase of a wave function oscillate with the spatial period of the potential, the parametric spin wave behaves in a similar manner in the time domain. Both situations can be mathematically described in terms of the Hill equation.

**Figure 6:**
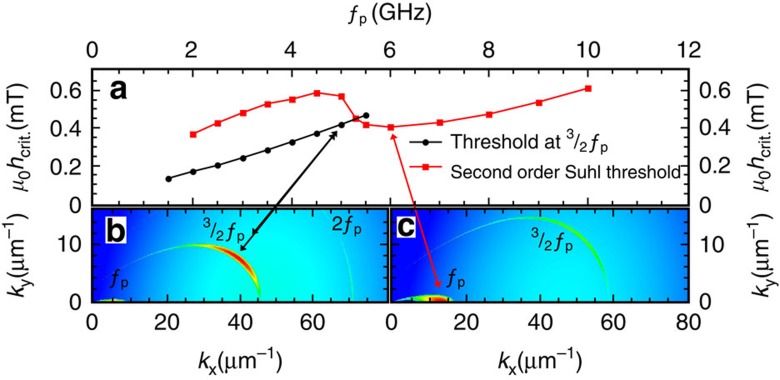
Calculation of the critical r.f. field amplitude as a function of the excitation frequency. (**a**) Above 6 GHz, the second-order Suhl instability becomes the relevant instability process with the lowest threshold. In the transition region, the second-order Suhl instability threshold sharply increases as a consequence of the positive nonlinear frequency shift for the critical spin waves. This frequency shift is more relevant at lower fields, because the spin-wave dispersion for *k*-vectors parallel to the magnetization direction is rather shallow at low fields (due to low resonance frequencies). This effect significantly decreases the *k*-vector of the critical spin wave and its coupling parameter with the uniform mode. This result is in excellent agreement with our experiments. In **b** and **c**, the transition between the low and high bias field instability processes is demonstrated. Here, the relaxation rates are shown as a function of wave vector for resonant pumping of the uniform modes with *f*_p_=5 GHz and *f*_p_=6 GHz, respectively. The simulation parameters were chosen to be as follows: *α*=0.009, *M*_S_=8 × 10^5^ A m^−1^ and film thickness 30 nm.

We would like to note that this type of behaviour has not been considered so far, and that it is worthwhile to examine previous experiments in the light of these findings. In particular, we believe that these nonlinear processes can explain the apparent wave-vector-dependent Gilbert damping reported by two groups under similar experimental conditions^27^,^28^,^29^. Reviewing the frequency-dependent measurements of nonlinear ferromagnetic resonance performed by Gerrits *et al.*^27^, we are able to accurately reproduce their experimentally found threshold fields with our model. In Fig. 6, corresponding calculations are shown for our sample thickness. Our simulations indicate that for frequencies below 5 GHz, spin waves oscillating at 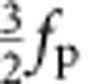 are parametrically excited before the second-order Suhl instability can set in (spin waves oscillating at *f*_p_). We therefore conclude that the observed threshold fields correspond to the type of instability processes described in the present work.

To verify the applicability of our model also for Suhl instability processes, the r.f. threshold amplitude fields are calculated as a function of magnetic bias field (so-called butterfly curves) and compared with published experimental results^30^ for subsidiary absorption (first-order Suhl instability) and for resonance saturation (second-order Suhl instability)^24^. We find very good agreement with the measurements in both cases using a wave-number-independent intrinsic Gilbert damping parameter. Furthermore, the nonlinear frequency shift is also observable in the butterfly curves of first-order Suhl instability thresholds^30^. According to our calculations, the frequency locking effect can explain why the experimental threshold fields in^30^ increased less abruptly above the resonance field than the authors expected.

We would like to point out that the validity of the above calculations is verified by extensive micromagnetic simulations^31^. As shown in the inset of Fig. 3 and by the points in Fig. 5a, the results of the micromagnetic simulation are in excellent agreement with our experimental results and our model description of the nonlinear dynamics. We also find that the micromagnetic simulations agree very well in terms of the threshold excitation field, the wave vector of the critical mode and the nonlinear frequency shift. An example of the spin-wave spectral density in *k*-space obtained from micromagnetic calculations is shown in Supplementary Fig. 4. In agreement with predictions from our model (see Fig. 4a)), the critical spin-wave modes actually oscillate non-harmonically at 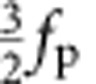 for low magnetic bias fields.

In conclusion, we investigate experimentally and theoretically the nonlinear magnetization dynamics in magnetic films at low magnetic bias fields. Our analysis leads to a new and more general description of parametric excitation not limited to small amplitudes of the modulation parameter. Using this method, we find a new class of spin-wave instabilities that dominate the nonlinear response at low magnetic fields. For these modes, we also find pronounced frequency locking effects that may be used for synchronization purposes in magnonic devices. By using this effect, effective spin-wave sources based on parametric spin-wave excitation may be realized. Our results also show that it is not required to invoke a wave vector-dependent damping parameter in the interpretation of nonlinear magnetic resonance experiments performed at low bias fields. The recipe that has been developed here should prove very valuable for the general description of nonlinear magnetization dynamics. Specifically, the model allows a fast prediction of the critical spin-wave modes.

## Methods

### Sample preparation

The samples are composed of a metallic film stack grown on top of a 100-nm-thick Si_3_N_4_ membrane supported by a silicon frame, to allow transmission of X-rays. The film system is patterned into a coplanar waveguide by lithography and lift-off processes. The nominal 40-nm-thick Ni_80_Fe_20_ layer is isolated from the 160-nm-thick copper layer by a 5-nm-thick film of Al_2_O_3_. The isolation layer and the low conductivity of Ni_80_Fe_20_ ensure that 95% of the r.f. current flow in the Cu layer, leading to a well-defined in-plane r.f. excitation of the sample. The driving field is oriented along the *y* direction perpendicular to the external d.c. field (transverse pumping).

### X-ray magnetic circular dichroism–FMR

XMCD is measured at the Fe L_3_ absorption edge. For circularly polarized X-rays, the dichroic component of the signal is proportional to the magnetization component along the X-ray beam direction. The size of the probed spot on the sample is defined by the width of the signal line of the coplanar waveguide structure (80 μm) and by the lateral dimension of the X-ray beam (900 μm). The transmitted X-ray intensity is detected by a photodiode^32^. All measurements are performed at the PM3 beamline of the synchrotron at the Helmholtz Zentrum Berlin in a dedicated XMCD chamber. The magnet configuration is shown in Supplementary Fig. 1. The microwave excitation in the XMCD–FMR experiment is phase synchronized with the bunch timing structure of the storage ring, so that stroboscopic measurements are sensitive to the phase of the magnetization precession. Synchronization is ensured by a synthesized microwave generator, which uses the ring frequency of 500 MHz as a reference, to generate the required r.f. frequency in the GHz range. The phase of the microwave excitation with respect to the X-ray pulses is adjusted by the signal generator. To allow for lock-in detection of the XMCD signal, the phase of the microwaves is modulated by 180° at a frequency of a few kHz. Owing to the synchronization of the microwave signal and the X-ray bunches, the magnetization is sampled at a given constant phase. The amplitude of the modulated intensity is proportional to the dynamic magnetization component projected onto the X-ray beam, as illustrated in Fig. 1. This signal is normalized by static XMCD hysteresis loops. The normalized signal is an absolute measure of the amplitude of the magnetization dynamics, evaluated in units of the saturation magnetization or as cone angle of the precession of the magnetization vector. Supplementary Fig. 2 shows typical XMCD measurements.

### Theoretical model

We start with the LLG equation in the following form:


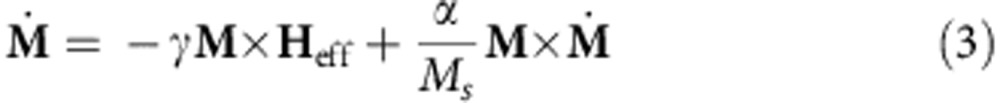


with 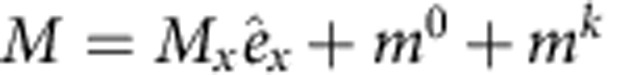, where we assume *M*_x_>*m*^0^>>*m*^*k*^, that is, the uniform precession *m*^0^ is smaller than the static uniform magnetization and the non-uniform dynamic magnetization *m*^*k*^ is much smaller than *m*^0^. *H*_eff_ is the effective field consisting of the external field, the exchange field (*h*_exch_=2*Ak*^2^/(*μ*_0_*M*_s_)) and the dipolar field (
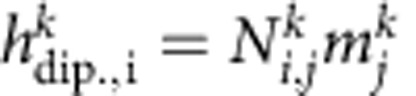
). Here we use a thin film approach^33^ for the dipolar tensor 
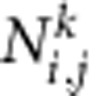
 instead of the more complicated expressions that we use for the analysis of parametric instability in thicker films^34^. To first order in the non-uniform spin-wave amplitudes, we find an equation of the form:


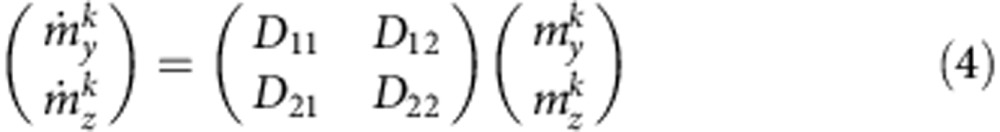


with time-dependent components *D*_*ij*_. The coupled coordinates can be separated by applying a time derivative. The result for 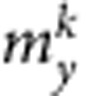 looks as follows (where we drop the superscript):


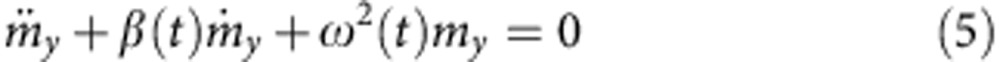


with 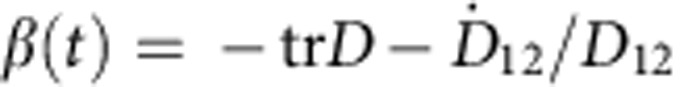 and 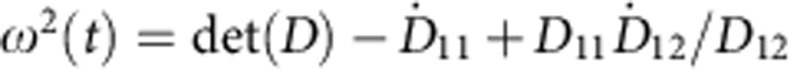. This form corresponds to the differential equation of a parametric oscillator. By substituting


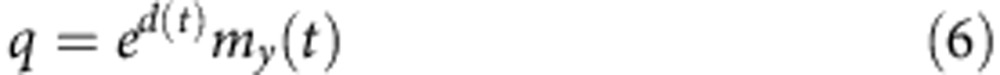


with 
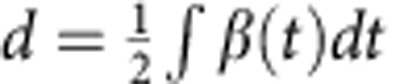
, we can eliminate the damping term:


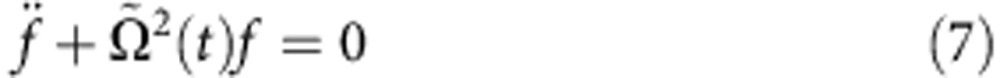


with 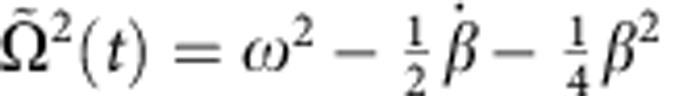. Now we introduce the dimensionless parameter *x*=*ω*_mod_*t*/2 and assume that 
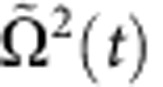
 varies periodically with the frequency *ω*_mod_. One thus obtains:


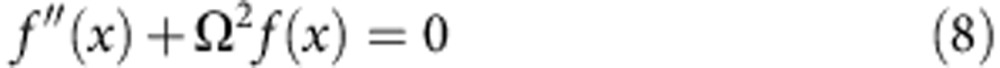


where we use 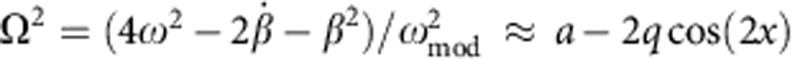, to find approximate solutions. This assumption only implies that the time-dependent modulation of the parametric oscillator is sinusoidal with a single frequency (for example, the driving frequency *ω*_*p*_). Equation (8) can then be written in the form of the Mathieu equation:


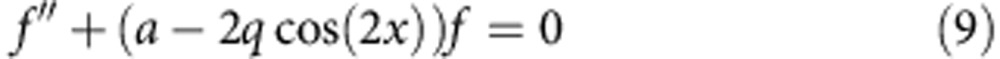


According to Floquet's theorem^35^, the solutions of this equation are of the form:


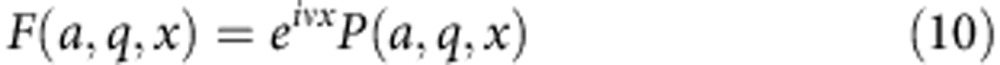


where the complex number *ν*=*ν*(*a*, *q*) is called the Mathieu exponent and *P* is a periodic function in *x* (with period *π*). The parameters *a* and *q* depend on the properties of the spin wave. From the knowledge of *ν* for a given *k*-vector, one can predict the behaviour of the corresponding spin wave as a function of time: For example, as soon as the imaginary part of 
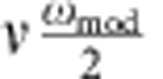
 exceeds the exponent in equation (6) the spin wave becomes critical. Furthermore, the real part of 
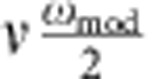
 corresponds to the frequency of the spin wave. This method can be used to quickly find the dispersion and the lifetimes of all possible spin-wave modes when the parameter *ν* is evaluated as a function of *k*_*x*_ and *k*_*y*_.

### Micromagnetic calculations

Micromagnetic simulations that confirm our conclusions are performed using an open source graphic processor unit-based code Mumax^31^. The simulated sample volume is 80 μm × 20 μm × 30 nm. Time traces of 500 ns are computed to extract the numerical values. Standard parameters for Ni_80_Fe_20_ are used in the simulations: saturation magnetization *M*_s_=8 × 10^5^ A m^−1^, damping constant *α*=0.009 and exchange constant *A*=13 × 10^−12^ J m^−1^. We find the best agreement between the simulations and the experiments for a Ni_80_Fe_20_ thickness of 30 nm, whereas in the experiments the nominal thickness of the Ni_80_Fe_20_ layer is 40 nm. We attribute this discrepancy mostly to the surface roughness of the Ni_80_Fe_20_ layer in the experiments.

## Additional Information

**How to cite this article**: Bauer, H. G. *et al.* Nonlinear spin-wave excitations at low magnetic bias fields. *Nat. Commun.* 6:8274 doi: 10.1038/ncomms9274 (2015).

## Supplementary Material

Supplementary Information Supplementary Figures 1-4 and Supplementary Reference
