# Supplementary material for: Nonlinear spin-wave excitations at low magnetic bias fields
Source: Nat Commun. 2015 Sep 16;6:8274. doi: 10.1038/ncomms9274 (PMC4595629; doi:10.1038/ncomms9274)
Supplement: Supplementary Information — Supplementary Figures 1-4 and Supplementary Reference [file ncomms9274-s1.pdf]

Supplementary Figure 1

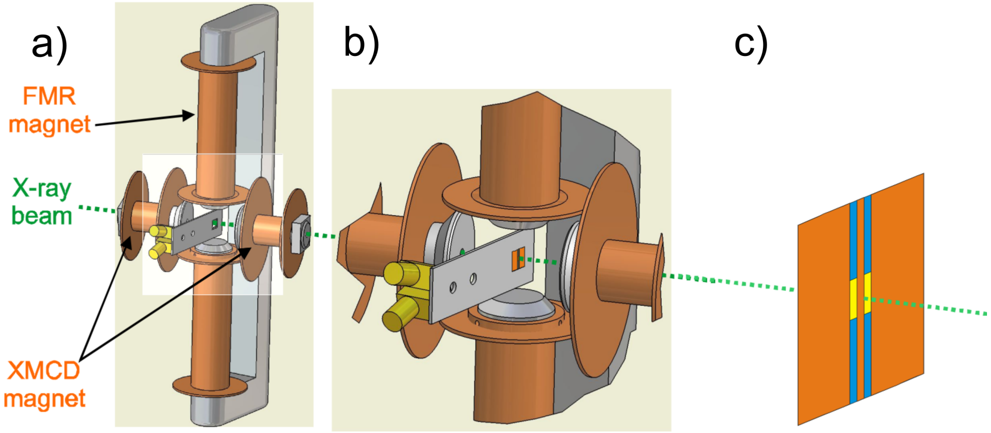

Supplementary Figure 1: The experimental setup. (a) The vacuum chamber is equipped with two electromagnets. The first magnet (XMCD magnet) is oriented in the X-ray beam direction and allows to measure static XMCD hysteresis loops in order to calibrate the XMCD signal from the FMR measurements. The second magnet (FMR magnet) generates the magnetic bias field for the FMR measurements perpendicular to the X-ray beam. **(b)** Shows a close up view of the magnet configuration with the sample holder mounted in the center. The sample holder can be tilted in any direction unsing a goniometer. **(c)** Magnified view of the layout of the sample with coplanar waveguide deposited on a Si_3N_4 membrane window substrate (silicon frame).

Supplementary Figure 2

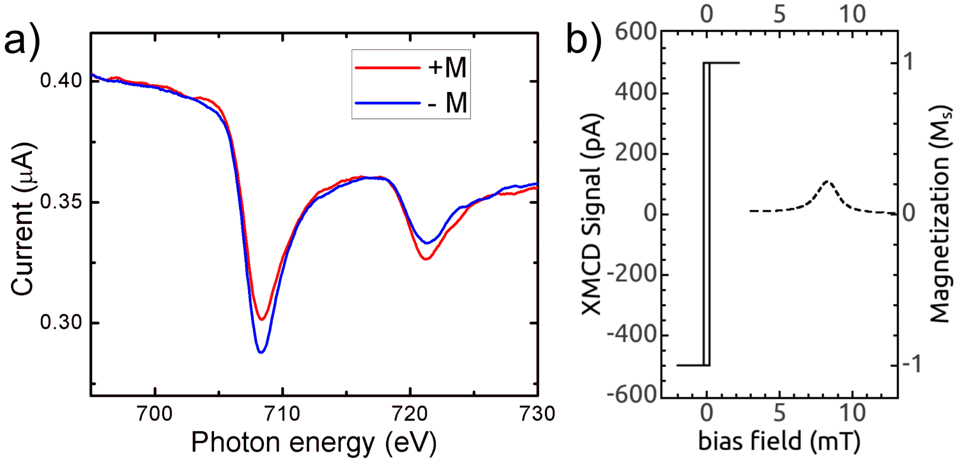

Supplementary Figure 2: **XMCD spectra.** **(a)** X-ray absorption spectrum of the L_{23} edge recorded for two in-plane orientations of the magnetization in a 40 nm thick $Ni_{80}Fe_{20}$ layer with the surface normal tilted by 20 degrees with respect to the X-ray beam. All dynamic (FMR) and static (hysteresis loops) measurements are performed at the X-ray energy where the dichroism is maximum (L_3 edge at 707 eV). **(b)** Normalized dynamic magnetization signal (dashed line). This signal was normalized to the saturation magnetization M_s, which is determined using static XMCD hysteresis loop recorded in the same setup (line).

Supplementary Figure 3

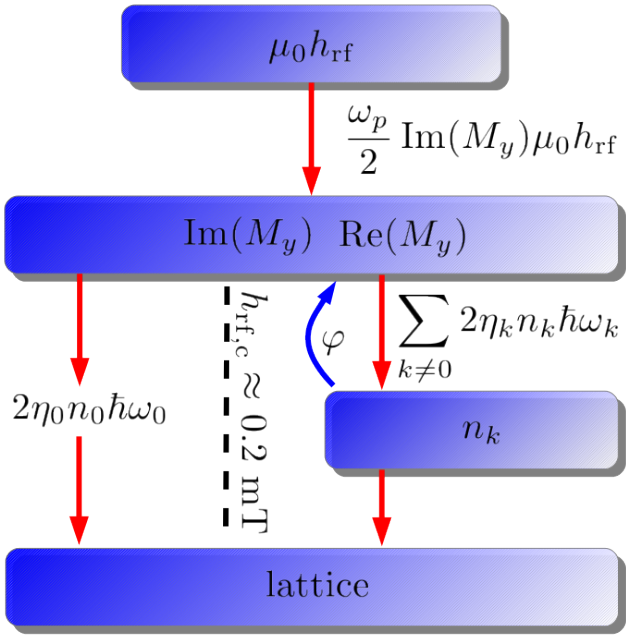

Supplementary Figure 3: **Diagram of the energy flow.** At first the energy flows from the driving field into the uniform precession mode which is detected by in-phase and out-of-phase components of the transverse in-plane magnetization ($Re(M_y)$ and $Im(M_y)$). Intrinsic Gilbert damping leads to relaxation of the energy from the uniform mode into the lattice. The corresponding relaxation time is determined by FMR measurements at low excitation amplitudes. Above the critical excitation field amplitude of about 0.2 mT non-uniform spin-wave modes are also excited. They react back on the uniform mode and cause a change of the phase angle ϕ between the uniform precession and the driving field. Due to Gilbert damping their energy is also relaxed into the lattice. Combined with the Gilbert damping of the uniform mode the relaxed energy must equal the energy pumped into the magnetic system. Since the energy pumped into the system and the Gilbert damping of the uniform mode are known precisely also the relaxation rate of the non-uniform spin-waves can be determined.

Supplementary Figure 4

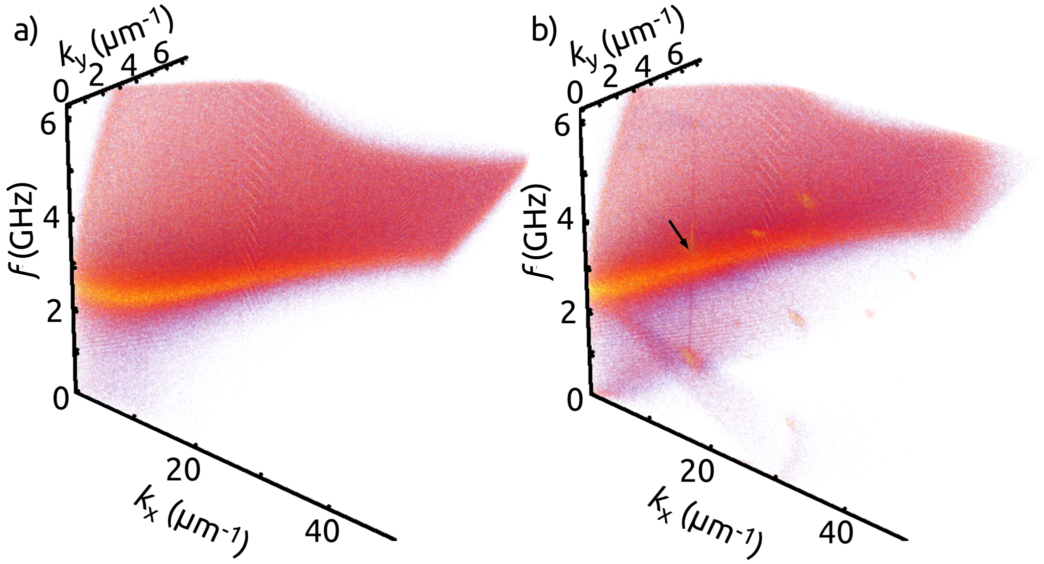

Supplementary Figure 4: **Micromagnetic simulation of the spin-wave density below and above the theshold amplitude.** The micromagnetic simulations are performed using the open source GPU based Mumax code [1]. Here a 1024×1024 cell grid with a large cell size of 100 nm×25 nm×30 nm is used in order to achieve a reasonable resolution in k-space. As the spin-waves for the considered range of wave vectors are dominated by the dipolar fields and the static magnetization is uniform the errors for the exchange contribution due to the large cell size are still acceptable. The results of the micromagnetic simulations are Fourier-transformed, representing the spin-wave density as a function of frequency in k-space. In **(a)** spin-waves are only excited by small random (thermal) fields at a bias field of $\mu_0 H_x = 8$ mT in order to visualize the linear spin-wave dispersion relation. In **(b)** an additional sinusoidal driving field of 2.5 GHz frequency and $\mu_0 H_y = 0.2$ mT amplitude is used to bring the system slightly above the spin-wave instability. One can see the critical spin-wave mode (marked with the arrow) and linear combinations of its k-vector at $f = \frac{3}{2} f_p$ and its sidebands at $f = \frac{1}{2} f_p$ and $f = \frac{5}{2} f_p$ as well as the upshift of the spin-wave manifold in k_x-direction.

References

[1] Vansteenkiste, A. *et al.* The design and verification of MuMax3. *AIP Advances* **4**, 107133 (2014). 1406.7635.
